# Application of Improved Sparrow Search Algorithm to Path Planning of Mobile Robots

**DOI:** 10.3390/biomimetics9060351

**Published:** 2024-06-11

**Authors:** Yong Xu, Bicong Sang, Yi Zhang

**Affiliations:** College of Electrical and Computer Science, Jilin Jianzhu University, Changchun 130119, China; xuyong@jlju.edu.cn (Y.X.);

**Keywords:** circle chaotic mapping, integration of northern goshawk exploration phase location strategy, adaptive T-distribution variation strategy, Lévy flight strategy, sparrow search algorithm, path planning

## Abstract

Path planning is an important research direction in the field of robotics; however, with the advancement of modern science and technology, the study of efficient, stable, and safe path-planning technology has become a realistic need in the field of robotics research. This paper introduces an improved sparrow search algorithm (ISSA) with a fusion strategy to further improve the ability to solve challenging tasks. First, the sparrow population is initialized using circle chaotic mapping to enhance diversity. Second, the location update formula of the northern goshawk is used in the exploration phase to replace the sparrow search algorithm’s location update formula in the security situation. This improves the discoverer model’s search breadth in the solution space and optimizes the problem-solving efficiency. Third, the algorithm adopts the Lévy flight strategy to improve the global optimization ability, so that the sparrow jumps out of the local optimum in the later stage of iteration. Finally, the adaptive T-distribution mutation strategy enhances the local exploration ability in late iterations, thus improving the sparrow search algorithm’s convergence speed. This was applied to the CEC2021 function set and compared with other standard intelligent optimization algorithms to test its performance. In addition, the ISSA was implemented in the path-planning problem of mobile robots. The comparative study shows that the proposed algorithm is superior to the SSA in terms of path length, running time, path optimality, and stability. The results show that the proposed method is more effective, robust, and feasible in mobile robot path planning.

## 1. Introduction

Mobile robot path-planning technology [1] allows the robot to plan a safe and efficient operation route through the information obtained by sensing the environment to complete the specified task. This process mainly deals with three kinds of problems: (1) Make the robot move from the initial to the target point. (2) Help the robot avoid obstacles and achieve corresponding tasks through necessary points. (3) Optimize the running trajectory of the robot as much as possible based on meeting the above requirements. Robot path-planning technology was developed as early as the 1970s, and it has accumulated rich research results that are an essential part of intelligent mobile robot research. Path-planning strategies [2] can be divided into three types from the perspective of environmental perception: environment model-based, case learning-based, and behavior-based. They can be divided into global and local path planning as far as the target category is concerned. In addition, they can also be divided into static path planning and dynamic path planning according to the time characteristics of the planning environment.

Path-planning technology represents a pivotal area of robotics research, as it is of paramount importance for enabling robots to navigate autonomously and perform intelligent tasks in unknown environments. During the operational phase, mobile robots must utilize sensors to gather environmental data for self-localization and environment map construction. Subsequently, they employ path-planning algorithms to identify the optimal collision-free path that fulfills specific criteria, such as the shortest effective path or the minimum energy consumption. However, in large-scale indoor warehousing environments, complex scenarios, dynamic noise, and other factors present higher requirements on the robot’s environment perception and autonomous navigation capabilities. It is not possible to simply apply existing path-planning techniques to this, necessitating in-depth research on the corresponding robotic path-planning techniques to address the special characteristics of the warehousing environment.

**Figure 1:**
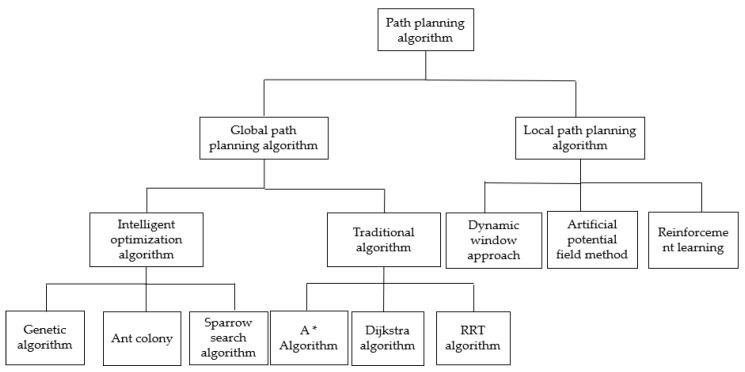
Classification of path-planning algorithms.

The core of path planning lies in algorithm design, and the path-planning algorithm [3] has been widely explored. It has gradually evolved from the initial traditional algorithm to an intelligent one combined with bionics. At present, the intelligent optimization algorithm has achieved remarkable results. All intelligent optimization algorithms have unique features, and their adaptive ranges and fields differ. Therefore, it is of great value to discuss the intelligent optimization algorithm of path planning from the perspective of the algorithm itself and its application category. Path-planning algorithms play a crucial role in many fields. A path-planning algorithm aims to find an optimal path to minimize costs or maximize benefits. They are widely used in transportation, robot navigation, drone flight, video games, and other fields. Path-planning algorithms can be classified according to different characteristics and needs and divided into graph-search-based and optimization-based algorithms. Graph-search-based algorithms build graph models and use search strategies to find the optimal path, such as Dijkstra’s algorithm [4] and the A* algorithm [5]. The path planning algorithms are categorized as shown in Figure 1. Optimization-based algorithms solve the optimal path by establishing mathematical models, such as the ant colony algorithm, genetic algorithm, particle swarm optimization algorithm, sparrow search algorithm, and other swarm intelligence algorithms. Swarm intelligence algorithms are widely used, especially in industrial optimization, path planning, and optimization calculation, because of their strong optimization ability and easy implementation. In these fields, the sparrow search algorithm is often used.

The swarm intelligence path-planning field has seen several advancements in recent years. Huang Zhanpeng et al. [6] proposed an improved ant colony optimization (IACO) algorithm that introduced local and global search mechanisms, thereby improving the performance of traditional ant colony algorithms in path planning. Zhou Xinghua et al. [7] proposed improved particle swarm optimization (IPSO), which introduced adaptive weights and dynamic parameter adjustment strategies to improve the search performance of traditional particle swarm optimization algorithms in path-planning problems. Li Ming et al. [8] proposed an improved genetic algorithm (IGA) that enhanced the search effect of the traditional GA in path-planning problems by introducing the improvement of cross-operation and an adaptive mutation strategy. Wang et al. [9] proposed a swarm intelligence path-planning algorithm based on genetic algorithms and deep learning, which optimized the parameters of the deep learning model through genetic algorithms to achieve more efficient path planning.

The sparrow search algorithm (SSA), inspired by the foraging and anti-predation behaviors of sparrow populations, is a relatively novel algorithm. Compared with other algorithms, the SSA has better stability and robustness, and it can be used for solving real-world problems, such as circuit problems, path planning, and TSP problems. However, to address the shortcomings of the sparrow search algorithm—such as premature convergence, weak global exploration ability, and weak ability to jump out of the local optimal solution, as well as the trade-off between the convergence speed and the population diversity in the optimization process—improvements are proposed in terms of the algorithm structure, the parameter control, and the path-planning strategy, to improve the algorithm’s ability to explore globally and locally, as well as its ability to jump out of the local optimum.

With the wide application of robot technology, all directions in the field of robotics have become hot research directions for modern science and technology, among which path planning is an important research direction in the field of robotics. However, traditional or single path-planning methods have been unable to cope with the increasing demand for robot applications. Therefore, it has become a realistic demand in the field of robot research to study efficient, stable, and safe path-planning technology. This paper studies how to use the sparrow search algorithm to solve such problems, as well as the difficulties and solutions in the application. This paper proposes an improved sparrow search algorithm (ISSA) for automated guided vehicle (AGV) path planning based on the abovementioned research. There are still considerable improvements to be made, although the methods proposed by the above research have improved the performance of swarm intelligence algorithms to some extent. Firstly, this improvement introduces the circle chaotic mapping function to initialize the population, enhancing the algorithm’s geotropism and improving its optimization ability. Secondly, we replace the position update formula of R_2_ < ST in the discoverer position update with the position update formula of the northern goshawk in the exploration phase to increase the exploration capability of the algorithm. Thirdly, we introduce the Lévy flight strategy into the follower update formula to improve the global search capability and prevent the algorithm from falling into local optimality. Lastly, we introduce T-distribution perturbation and variation with a certain probability in the follower stage of the sparrow algorithm to speed up the algorithm’s convergence rate. The performance of the proposed algorithm was tested on the CEC2021 function set and compared with the results of common intelligent optimization algorithms. The ISSA was applied to the path-planning problem of mobile robots. The simulation was carried out with a fixed-obstacle environment map and random-obstacle environment map, respectively, and compared with the planning paths of the GA, PSO, DE, GWO, and SSA. The simulation results show that, compared with other algorithms, the ISSA not only shortens the path optimization time and shortens the path length of the mobile robot, but also reduces the number of path turns and improves the smoothness of the path. The results show that the proposed method is more effective, robust, and feasible in mobile robot path planning.

The structure of this paper is as follows: Section 1 explains the theory and mathematical model of the sparrow search algorithm. Section 2 introduces the circle chaotic mapping function to enhance the algorithm’s credibility and the Lévy flight strategy to improve its global search capability. In Section 3, the practicability of the ISSA is verified through comparison with other algorithms. Section 4 uses an improved sparrow search algorithm for global path planning. Section 5 summarizes the research and points to future directions.

## 2. Sparrow Search Algorithm

The sparrow search algorithm [10] is a heuristic search algorithm for optimization problems. The behavior of sparrows inspired it in their search for food. The sparrow search algorithm simulates the sparrow’s strategy when searching for food, searching for the optimal solution by constantly foraging and exploring new areas. In the traditional sparrow search algorithm, the position of each sparrow can be expressed as follows for a problem with D-dimensional solution space: Population behavior is mainly divided into three modes during the optimization stage. For a problem with a D-th solution, the space position of every sparrow can be expressed as $X_{i}=(x_{i,1},x_{i,2},x_{i,3},\cdots ,x_{i,D})$, namely, discoverer, followers, and early warning, where the discoverer’s update location can be expressed as follows:

(1) $$X_{i,j}^{t+1}=\{\begin{array}{cc} X_{i,j}^{t}\cdot \exp\;\left(\frac{-i}{\alpha \cdot T}\right) & \;\mathrm{if}\;R_{2}<ST\\ X_{i,j}^{t}+Q\cdot L & \;\mathrm{if}\;R_{2}\geq ST \end{array}$$

where t represents the current number of iterations, α is a random number in the range (0, 1), *R*_2_ ∈ (0, 1) represents the current alert value, ST ∈ (0.5, 1) represents the safety threshold, and Q is a random number that follows a normal distribution. L is a 1 × d matrix, where every element is 1. When *R*_2_ < *ST*, it means that the danger level in the current environment is not high, and the discoverer can search for food in a large area. In contrast, when the alarm value exceeds the safety threshold (*R*_2_ ≥ *ST*), the sparrow population is aware of the danger, and the discoverers stop foraging in a large area, move to a safe location to avoid the threat, and search a small area at the same time.

The number of discoverers in the sparrow population is usually about 10–20%, and the rest act as followers. Followers always observe other individuals in the group and follow the individual they think is better, waiting for an opportunity to compete for food. Otherwise, they need to fly somewhere else to forage. The specific update formula for followers is as follows:

(2) $$X_{i}^{t+1}=\{\begin{array}{cc} Q\times exp\;(\frac{X_{worst}^{t}-X_{i}^{t}}{i^{2}}), & i>\frac{n}{2}\\ X_{p}^{t}+|X_{i}^{t}-X_{p}^{t}|\times A^{+}\times L, & i⩽\frac{n}{2} \end{array}$$

where *X*_worst_ represents the current global worst position and *X*_p_ is the optimal position occupied by the current finder. Parameter A represents a 1 × d matrix, all elements of which are 1 or −1, and A+ = (AAT) – 1. When i > n/2, it indicates that the i th follower with a low fitness value is in a poor state and needs to fly to other places for food. Otherwise, it follows the discoverers who follow the current best and searches for vegetarian foods near them for better food. It is assumed that 10–20% of the sparrow population will be aware of the danger and are called watchmen. The initial positions of these sparrows are randomly generated within the population. This can be expressed as follows:

(3) $$X_{i,j}^{t+1}=\{\begin{array}{cc} X_{\mathrm{best}\;}^{t}+\beta \cdot |X_{i,j}^{t}-X_{\mathrm{best}\;}^{t}| & \;\mathrm{if}\;f_{i}>f_{g}\\ X_{i,j}^{t}+K\cdot (\frac{\left|X_{i,j}^{t}-X_{\mathrm{worst}\;}^{t}\right|}{(f_{i}-f_{\mathrm{worst}\;})+\varepsilon }) & \;\mathrm{if}\;f_{i}=f_{g} \end{array}$$

where *X* represents the current global best position, *β* is a random number conforming to the standard normal distribution, K is a random number uniformly distributed in the range [−1, 1], *f*_i_, *f*_g_, and *f*_w_ represent the fitness value of the ith individual and the global best and worst fitness values in the current population, respectively, and *ε* is the smallest constant to avoid having a denominator of zero. When *f*_i_ > *f*_g_, it indicates that the individual sparrow is in a poor position in the population and needs to move towards a better position in the population to avoid danger. A value of *f*_i_ = *f*_g_ indicates that the superior sparrow has sensed danger and needs to move closer to other individuals to reduce the risk of predation.

The following pseudo-code (Algorithm 1) represents the specific algorithm flow according to the algorithm design steps.
**Algorithm 1** The framework of the SSA**Input**: T: the maximum iterations *num*_p_: the number of producers *num*s: the number of sparrows who perceive the danger R_2_: the alarm value pop: the number of sparrows Initialize a population of n sparrows and define its relevant parameters. **Output**: X_best_, f_g_. 1: **while** (t < T) 2: Rank the fitness values and find the current best individual and the current worst individual. 3: R_2_ = rand(1) 4: **for** i = 1: *num*_p_ 5: Using Equation (1) update the sparrow’s location; 6: **end for** 7: **for** i = (*num*_p_ + 1): pop 8: Using Equation (2) update the sparrow’s location; 9: **end for** 10: **for** l = 1: *num*_s_ 11: Using Equation (3) update the sparrow’s location; 12: **end for** 13: Get the current new location; 14: If the new location is better than before, update it; 15: t = t + 1 16: **end while** 17: **return** X_best_, f_g_.

## 3. Improved Sparrow Search Algorithm

### 3.1. Circular Chaotic Mapping

While the SSA performs well in many optimization problems, it also has some drawbacks. First of all, the SSA relies on random behavior, which makes the search process of the algorithm highly random, which may cause the algorithm to oscillate around the optimal solution and reduce the search efficiency. Secondly, some parameters in the SSA, such as population size, update frequency, and pheromone importance, impact the algorithm’s performance, increasing its complexity and difficulty. In addition, the SSA can fall into local optimality, especially if the search space is complex or the problem has multiple local optimal solutions. It can converge more slowly than other heuristic algorithms, while the SSA converges quickly in many cases.

**Figure 2:**
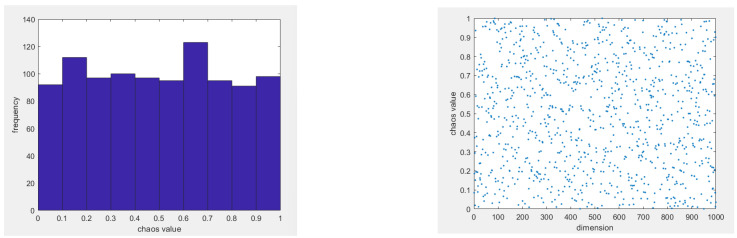
Circle mapping.

We can use chaotic operators to initialize the population to enhance the algorithm’s global search ability and avoid decreasing the population diversity. Chaos operators have the characteristics of randomness and regularity, and they can traverse all states in a particular range without repeating. Therefore, this paper proposes referencing circle mapping in the population initialization stage. Compared with logistic mapping [11], circle mapping [12] has a more extensive period range, decadent dynamic behavior, insensitivity to initial conditions, and better uniformity. These features make circle mapping more valuable in some applications. The mathematical expression of the circle map is as follows:

(4) $$z_{k+1}=z_{k}+a-mod\left(\frac{b}{2\pi }\sin\left(2\pi z_{k}\right),1\right)$$

where a = 0.5, b = 2.2, and mod in the circle expression is the complementary function. From Figure 2, it can be seen that the circular map is distributed between [0, 1], and its chaotic nature replaces the random initialization, resulting in a more uniform distribution of the population in the search space. We increase the sparrow population’s size and enhance its diversity by using circle chaotic mapping to construct the initial population of sparrows. Compared with those sparrows generated by random strategies only, those based on circle chaotic mapping are more evenly distributed and show higher population diversity. This mapping method can effectively reduce the accumulation of sparrow populations in marginal areas and achieve a more uniform distribution in non-marginal areas. This change expands the algorithm’s search space and significantly improves its search efficiency.

### 3.2. Integration of Northern Goshawk Exploration Phase Location Strategy

In the basic SSA, each dimension of the individual sparrow becomes smaller when R_2_ < ST, the discoverer decreases with the number of iterations, the search space gradually decreases, and the probability of falling into the local space increases. The northern eagle algorithm is able to search the solution space efficiently and approach the optimal solution step by step, with the advantages of diversity exploration, fast convergence, and global optimization capability in the exploration phase. This makes it an effective algorithm for solving complex optimization problems. The position update formula of R_2_ < ST during the discoverer position update is replaced by the position update formula of the northern goshawk [13] during the exploration phase to improve the adequacy of the discoverer model in solution space search and its performance in solving optimization problems. The learning factor formula and the improved discoverer position formula are as follows:

(5) $$x_{i,j}^{new,P1}=\{\begin{array}{c} x_{i,j}+r\left(p_{i,j}-Ix_{i,j}\right),F_{P_{i}}<F_{i}\\ x_{i,j}+r\left(x_{i,j}-p_{i,j}\right),F_{P_{i}}>F_{i} \end{array}$$

where $P_{i}=X_{k},i=1,2,\ldots ,N,k=1,2,\ldots ,i-1,i+1,\ldots ,N$

### 3.3. Lévy Flight Strategy

The advantages of the Lévy flight strategy lie in its search diversity and long jump properties, which can effectively avoid local optimal solutions and explore the whole search space. At the same time, it has better convergence performance and robustness, and it is suitable for different types of optimization problems.

The follower becomes the discoverer when the discoverer iterates for a certain number of times, and the fitness value remains unchanged. The Lévy flight strategy [14] is introduced into the follower update formula to improve the global search capability and avoid the algorithm falling into local optimality. The improved formula is as follows:

(6) $$X_{i,j}^{t+1}=\{\begin{array}{cc} Q\cdot exp\;(\frac{X_{\mathrm{worst}\;}^{t}-X_{i,j}^{t}}{i^{2}}) & i>\frac{n}{2}\\ X_{p}^{t+1}+X_{p}^{t+1}⊗Levy\;(d) & \;\mathrm{other}\; \end{array}$$

In Formula (7), $X_{p}^{t+1}$ is the best position occupied by the current discoverer, and the Lévy flight mechanism is as follows:

(7) $$Levy\;(x)=0.01\times \frac{r_{3}\times \sigma }{{\left|r_{4}\right|}^{\left(1/\xi \right)}}$$

In Formula (8), r_3_ and r_4_ are random numbers in the range of [0, 1], and ξ can be 1.5; σ is calculated as follows:

(8) $$\sigma ={\left(\frac{\Gamma (1+\xi )\times sin\;(\pi \xi /2)}{\Gamma ((1+\xi )/2)\times \xi \times 2^{\left(\xi -1)/2\right)}}\right)}^{\left(1/\xi \right)}$$

where Γ(x)=(x−1)!

### 3.4. Adaptive T-Distribution Variation Strategy

In the follower stage of the sparrow algorithm, T-distribution perturbation and variation with a certain probability [15] will not change the updating principle formula of the original sparrow algorithm, so the sparrow algorithm has a good global development ability in the early iteration stage and an excellent local exploration ability in the later iteration stage, thus speeding up the convergence speed of the sparrow algorithm.

(9) $$X_{\mathrm{new}\;}^{j}=X_{\mathrm{best}\;}^{j}+t(C\_\mathrm{iter})\cdot X_{\mathrm{best}\;}^{j}$$

where t(C_iter) is a variant, $X_{\mathrm{new}\;}^{j}$ represents the position of the individual sparrow after mutation, $X_{\mathrm{best}\;}^{j}$ represents the best position of the individual sparrow, and t(C_iter) represents the T-distribution with the number of iterations, which is regarded as the degree of freedom parameter. Based on this definition, the random disturbance term of T-distribution is introduced, the information disturbance of the current population is fully utilized, and the iteration number t is set as the freedom parameter. The number of initial iterations is moderate, and the variation of T-distribution is similar to that of the Cauchy distribution, which has strong global search performance. The later stage resembles Gaussian distribution variation, with excellent local development potential. In the middle stage of the algorithm, the T-distribution variation is between Cauchy variation and Gaussian variation. The T-distribution mutation operator has the advantages of Gaussian and Cauchy operators, and it optimizes the algorithm’s global and local exploration performance.

The following pseudo-code (Algorithm 2) represents the specific algorithm flow according to the above algorithm design steps.
**Algorithm 2** Improved sparrow search algorithm (ISSA)**Input**: T: the maximum iterations *num*_p_: the number of producers *num*s: the number of sparrows who perceive the danger R_2_: the alarm value pop: the number of sparrows Circle maps the Halton sequence to initialize the sparrow population pop and define relevant parameters; **Output**: X_best_, f_g_. 1: **while** (t < T) 2: Rank the fitness values and find the current best individual and the current worst individual. 3: R_2_ = rand(1) 4: **for** i = 1: *num*_p_ 5: Using Equation (5) update the sparrow’s location; 6: **end for** 7: **for** i = (*num*_p_ + 1): pop 8: Using Equation (6) update the sparrow’s location; 9: **end for** 10: **for** l = 1: *num*_s_ 11: Using Equation (3) update the sparrow’s location; 12: **end for** 13:If rand < p, adaptive t-distribution mutation is performed according to Formula (9),the current optimal value is disturbed, and a new solution is generated. 14: Get the current new location; 15: Determine whether the conditions are met and output the results if they are met. Otherwise, repeat the 2 until the end condition is met.; 16: t = t + 1 17: **end while** 18: **return** X_best_, f_g_.

## 4. Algorithm Performance Test

### 4.1. Algorithm Parameter Settings

We selected particle swarm optimization (PSO) [16], Harris hawk optimization (HHO) [17], dung beetle optimization algorithm (DBO) [18], Young’s double-slit experiment optimizer (YDSE) [19], gray wolf optimization algorithm (GWO) [20], goshawk optimization algorithm (NGO) [21], and sparrow search algorithm (SSA) as comparison objects to verify the practicability of the ISSA and its influence on algorithm performance. The setting of relevant parameters of each algorithm is listed in Table 1.

### 4.2. Comparative Analysis of Algorithm Performance

**Figure 3:**
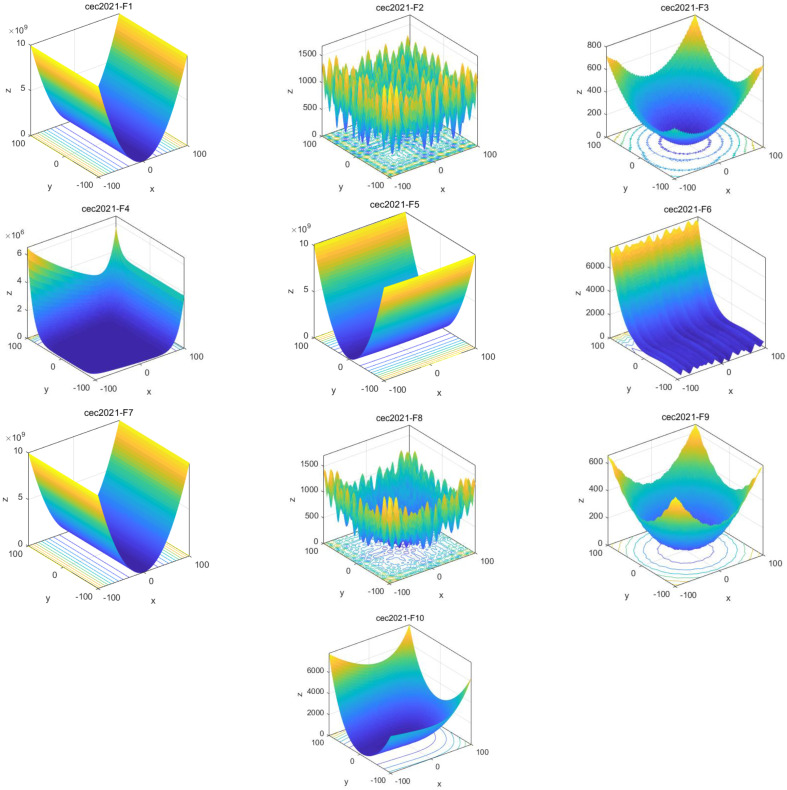
Ten images of test functions.

In this thesis, we rigorously tested and evaluated the performance of different algorithms based on the test function cec2021 [22], as shown in Table 2. Figure 3 shows the 2D shape of the cec2021 test function. In order to increase the fairness of the comparative experiments, we used Matlab2017 b to write the algorithm and simulate it. The algorithm was run on a Windows 10 64-bit system with 8 GB memory. The standard test functions were divided into four groups: unimodal test function (F1), basic function (F2–F4), mixed function (F5–F7), and combined function (F8–F10), as shown in Table 2. In the field of swarm intelligence optimization algorithms, this is often used to evaluate feasibility, efficiency, and robustness.

In order to ensure the accuracy of the test results and reduce the possibility of errors, we ran each test function 30 times to ensure reliable results. All algorithms used the same population size and number of iterations, that is, the number of populations was set to N = 30 in the test experiments, the maximum number of iterations was T = 1000, and the dimension was D = 10 to ensure reliable results. The measurement indices were analyzed and compared, including the optimal value, the worst value, the average value, the median, and the variance. Through the analysis of the results, we can draw the conclusions shown in Table 3.

The results of different algorithms for solving the CEC 2021 test suite functions are shown in Table 3. The results show that the ISSA works well on the 10 test functions. Obviously, the solution accuracy of the ISSA, which is optimized and improved compared to the original SSA, is greatly improved. The ISSA can converge to the global minimum in the other nine functions, except that it does not reach the optimal solution in the hybrid function F7, which fully demonstrates that our proposed algorithm has the ability to search globally. In the hybrid function F8, six algorithms (ISSA, SSA, DBO, GWO, NGO, and HHO) can find the global optimal solution, which is better than the other algorithms. The experimental results show that the advantage of the WNGO algorithm is obvious and its competitiveness is significantly better than that of the other seven algorithms. For each test function, the average value of the ISSA’s solution is close to the global optimum, with a relatively low standard deviation. This situation indicates that the fluctuation of the solution data is low and the optimization process of the algorithm shows more reliable stability. Therefore, compared with other algorithms, such as the SSA, DBO, NGO, GWO, PSO, YDSE, and HHO, the optimization performance of the ISSA is more accurate, and it can more easily find the optimal solution.

#### 4.2.1. Comparative Analysis of Algorithm Convergence Curves and Boxplots

**Figure 4:**
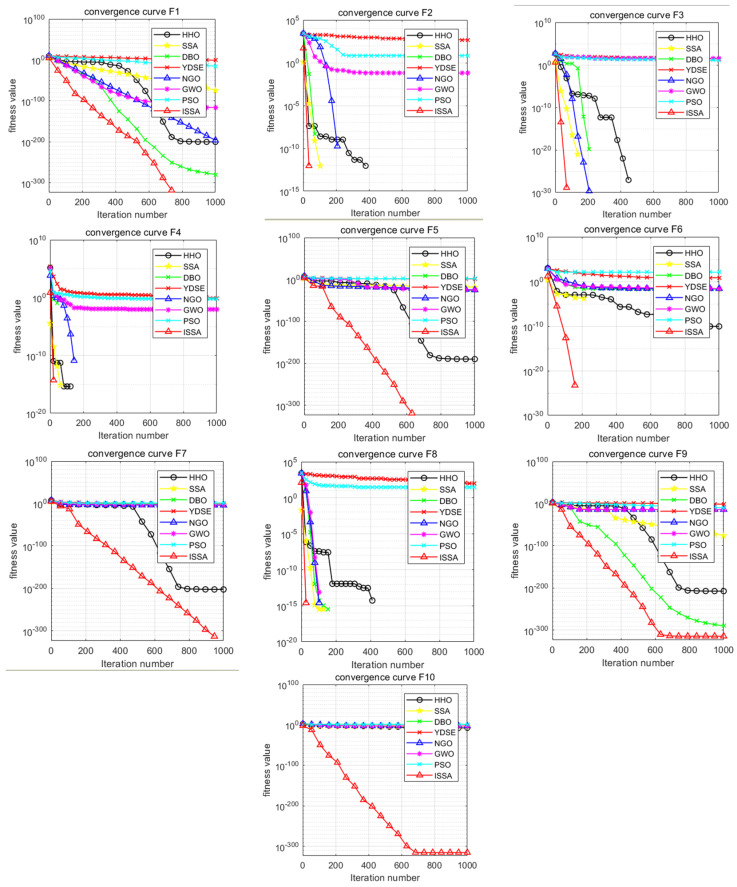
Convergence curves of 10 benchmark functions.

In order to intuitively compare the convergence speed of these eight algorithms, the convergence curves of the unimodal test function (F1), the basic functions (F2–F4), the mixed functions (F5–F7), and the combined functions (F8–F10) are shown in Figure 4. It can be seen that the ISSA has an absolute advantage in the convergence speed on all 10 test functions, making it significantly better than HHO, SSA, DBO, YDSE, GWO, PSO and NGO. On the test function, the ISSA can converge to the optimal value faster than the other seven algorithms. According to the convergence curve, the ISSA obtained a better fitness value in the initial stage, and it can be concluded that the ISSA has a certain competitiveness compared with the other seven algorithms. The smoothness and stability of the convergence curve are essential indicators for evaluating the algorithm’s performance. The convergence curve of the ISSA shows a good convergence trend, which indicates that the ISSA is stable in gradually approaching the target value. Compared with the other five algorithms, the curve of the ISSA is always kept at the bottom, which indicates that its convergence speed is faster, and the smaller the corresponding value, the higher the accuracy of its solution.

**Figure 5:**
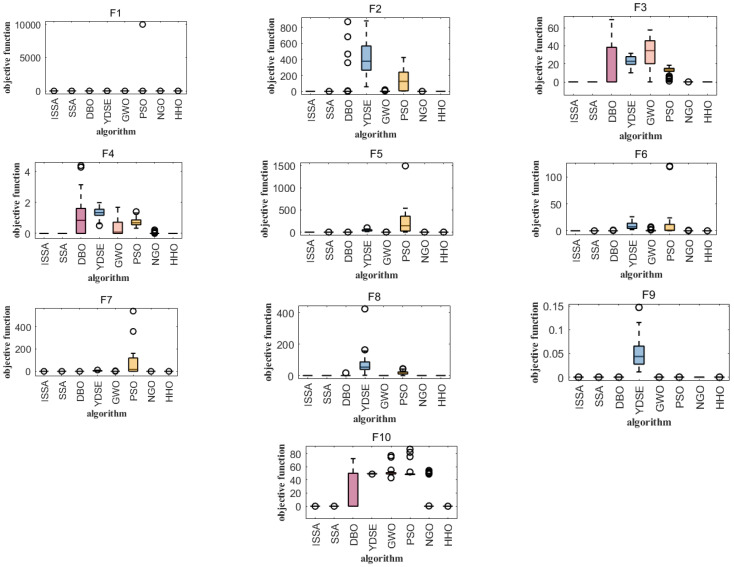
Comparison of 10 test function boxplots.

Boxplot analysis was carried out to compare the uniformity performance for all of the considered algorithms [23]. Boxplot analysis tools can effectively graphically represent the empirical distribution of data. Figure 5 shows the boxplots of mean functional assessments for the ISSA, PSO, SSA, DBO, NGO, YDSE, HHO, and GWO. It is clear from Figure 5 that the ISSA is cost-effective in terms of functional evaluation, as it has a smaller range of quartiles and a lower median of the mean functional assessment. On the unimodal test function (F1), the basic functions (F2–F4), the mixed functions (F5–F7), and the combined functions (F8–F10), the box length of the ISSA is smaller than that of the original SSA and other algorithms, indicating that the distribution of the results of the ISSA is more concentrated after running 30 times on these functions. It can be clearly seen from Figure 5 that the ISSA is cost-effective in functional evaluation, because the quartile range of the ISSA is small and its median of average functional evaluation is low. When comparing the algorithms considered according to the standard deviation and success rate, it can be seen from F2, F3, and F10 in Figure 5 that the performance of YDSE and DBO is relatively poor, while the PSO algorithm performs poorly in the test functions F2–F7. It can be seen that the ISSA, SSA, NGO, and HHO perform better in the test function CEC2021.

#### 4.2.2. Compared with the Improved Sparrow Algorithm (CASSA), Which Combines Cauchy Mutation and Opposition-Based Learning

**Figure 6:**
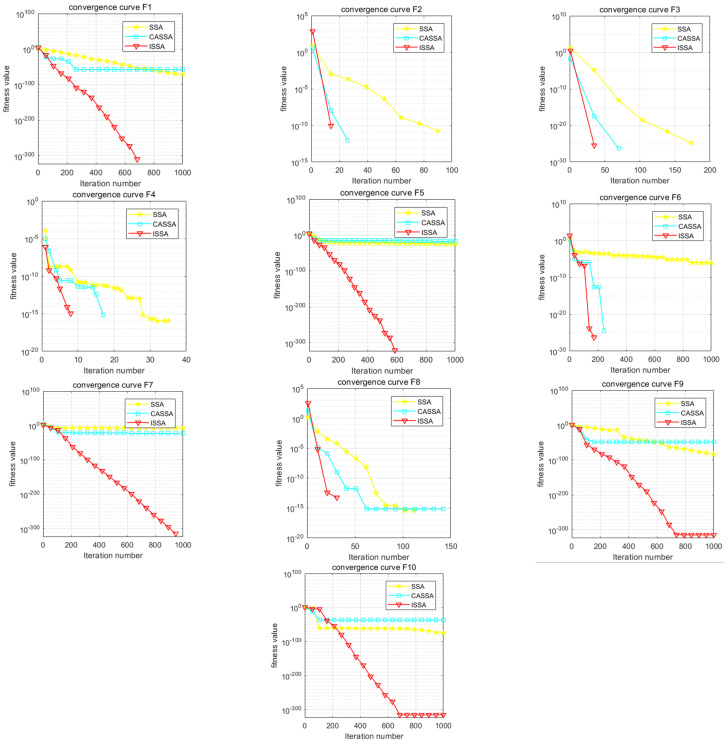
Convergence curves of 10 test functions for three algorithms.

In order to further reflect the superiority of the improved sparrow search algorithm, the performance of the improved sparrow algorithm (CASSA) [24] and SSA combined with Cauchy mutation and reverse learning was compared through 10 test functions of CEC2021. The general conditions were based on the literature. In the test experiment, the population number was set to N = 30, the maximum number of iterations was T = 1000, the dimension was D = 10, and the number of runs was 30. The comparison results are shown in Table 4 and Figure 6.

The comparison results show that the convergence curve of the ISSA is smoother than that of the SSA and CASSA, and the ISSA curve is always at the bottom, which indicates that the convergence speed is faster, while the smaller logarithmic value indicates that the solution accuracy is higher. In the F2, F3, F6, and F8 functions, the convergence speed of the ISSA and CASSA is obviously faster, while in the F5, F9, and F10 functions, the effect of the convergence curves of the CASSA is worse than that of the SSA, Table 4 lists the results of simulation experiments of the SSA, ISSA, and CASSA for the 10 test functions of CEC2021. From Table 4, it can be seen that the mean and standard deviation reflect the solution quality of the SSA, ISSA, and CASSA for the test functions F1 to F10, and it can be concluded that the ISSA is better than the other algorithms. In the test functions F3, F4, and F8, all three algorithms achieve the global nearest solution, with excellent results. The standard deviation reflects the robustness and stability of the algorithms, and the advantage of the ISSA is more obvious for functions F1 to F10.The optimal value of the ISSA is the best result obtained by the algorithm in a series of test functions. This reflects the maximum potential and capability of the algorithm. In terms of solving a single problem, the mean and standard deviation of the ISSA are small, which indicates that the ISSA is a more efficient algorithm with high accuracy and stability in the solving process. 

**Figure 7:**
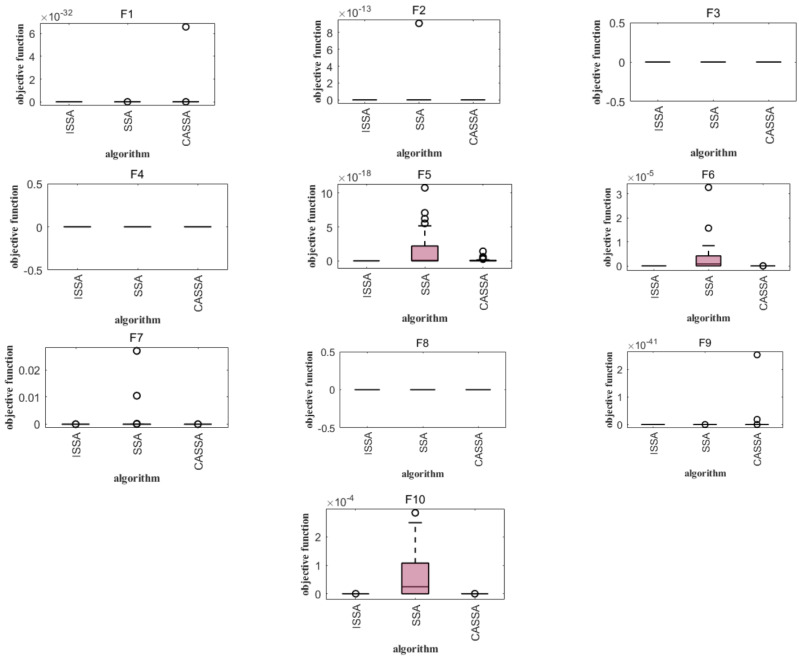
Comparison of 12 test function boxplots.

Analyzing Figure 6 and Figure 7, it can be seen that, compared with the same type of SSA and CASSA, the median value obtained by the ISSA is always close to the optimal value of each test function, and the change in the optimal value is minimized after iterative computation; in particular, the distribution of the solutions of the ISSA is more centralized, which demonstrates the superior robustness of the ISSA. The CASSA and ISSA cannot improve their optimization performance for these benchmark functions. In the other tested functions, the ISSA has significantly fewer outlier points than the other two algorithms. In F3, although the other two algorithms do not have outliers, the optimal value found by the ISSA has a smaller range of variation, which proves that it is more stable; in F5, the CASSA outperforms the traditional SSA, with a slightly lower convergence accuracy than the ISSA, but with a lower time complexity. The ISSA improves the convergence speed and search accuracy with very little change in time complexity, and regardless of the single-peak test function (F1), the basis functions (F2–F4), the hybrid functions (F5–F7), and the combination functions (F8–F10), the box lengths of the ISSA algorithm are smaller than those of the original SSA and the CASSA, which indicates that the ISSA has a more centralized distribution of results after 30 runs on these functions. Accordingly, the ISSA is expected to expand the search space of the algorithm, improve the search accuracy, and achieve an effective balance between global search and local optimization.

#### 4.2.3. Ablation Experiment

The ISSA is an algorithm with good optimization performance, and its advantages are attributed to a variety of additive mechanisms. However, it is not clear which additive mechanisms play a major role and whether there are certain additive mechanisms that do not work [25]. In order to more accurately understand the roles of various mechanisms, as well as to make the experimental results more credible, we set the CirSSA to contain only the circle mapping mechanism, the LeySSA to contain only the Lévy flight mechanism, the NGOSSA to incorporate the mechanism of the northern hawk algorithm, and the ALSSA to contain only the mechanism of adaptive T-distribution variability. Five standard test functions were selected for optimization of CEC2021 (F3, F4, F6, F8, and F9), and the other parameters were the same as in Section 4.1; the experimental results are shown in the following figure.

**Figure 8:**
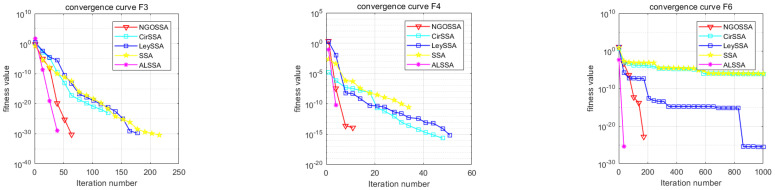

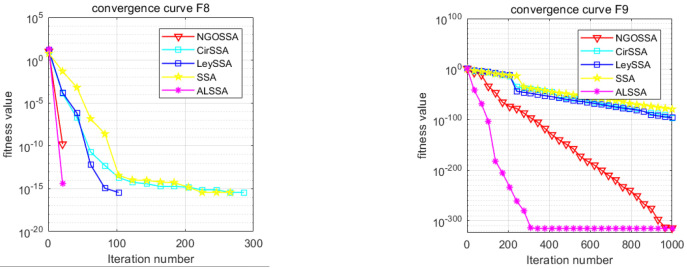
Ablation experiment results’ convergence curves.

**Figure 9:**
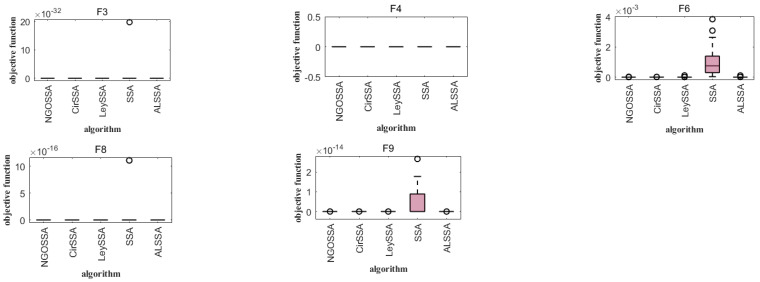
Ablation experiment results’ boxplots.

It can be seen from Table 5 that the four improvements in this paper improved the SSA, and the performance increased to varying degrees, indicating that the improved contents had a positive effect on the algorithm. Among them, for the function F4, because the SSA reached the global optimal solution, other improvement effects did not show specific performance. As can be seen in Figure 8 and Figure 9, the four improved strategies to improve the SSA showed good results in these five test functions. In particular, the adaptive T-distribution mutation strategy of the ALSSA performed best, while for the circle mapping mechanism of the CirSSA, although it also had a positive effect, the effect was not obvious. In summary, the ablation experiment shows that the four improved strategies of the ISSA are reasonable and effective.

## 5. Mobile Robot Path Planning

The simulation experiment was carried out using Matlab R2020b software. The computer operating system was Windows 10, Intel (R) Core (TM) i5-4200H CPU @ 2.80GHZ, and the memory was 8 GB. In this study, the planar grid environments with simulation map scales of 20 × 20, 30 × 30, and 40 × 40 were tested.

### 5.1. Environmental Model Map Building

The research object of this paper is to find the best path in a given environment. We treat the robot as a particle to simplify the problem. We must perform the obstacle expansion process to ensure the safe passage of the mobile robot in the working environment. In this part, we use the grid method [26] to divide the robot’s workspace into square grid cells of equal size. In practical application, the choice of mesh size is critical; the smaller the grid, the more accurate the description of obstacles, but the required storage space will also increase accordingly. As shown in Figure 8, the grid affected by obstacles [27] is called a free grid (marked in white), while the grid with obstacles is called an obstacle grid (marked in purple).

**Figure 10:**
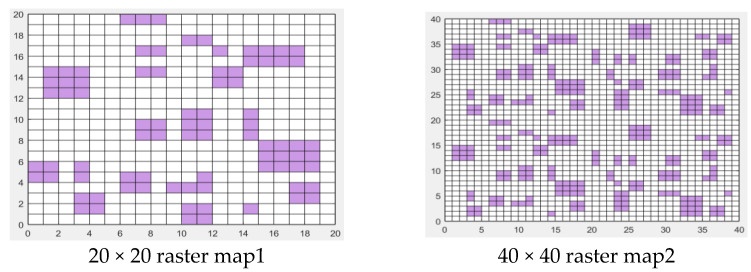
Map environment model.

Two map environment models were constructed to simulate the environment around the mobile robot, as shown in Figure 10. The horizontal and vertical axes correspond to the width and length of the map model, respectively. The map side length of environment model 1 is 20 × 20, the origin represents the starting point of the mobile robot, and the coordinate is (1, 1). The top right corner is the target point, with coordinates (20, 20). The map side length of environment model 2 is 40 × 40, the origin is the starting point, and the coordinate is (1, 1). The target points are (40, 40). By comparison, the environment model in Figure 10 map2 is relatively more complex, which will bring particular challenges to the path planning of mobile robots.

### 5.2. Establishment of the Random-Obstacle Environment Map

The core element of constructing an environment model is that it be easy for computers to store, process, and use. In path planning, geometric and raster methods are commonly used in graphical environment modeling, and raster maps are the most widely used processing and expression methods. Grid maps treat the environment as a network of square grids with a specific resolution, independent of each other. They can be divided into free and occupied states depending on whether obstacles occupy them. Raster maps are easy to construct and implement path planning with, and they are prevalent in robotics and autonomous driving. The grid of a map contains two critical factors: the size of the map, and the number of obstacles. Maps should be large enough to cover accessible navigation areas but as small as possible to improve storage and computing efficiency. It is clear that, at a given map size, the number of grids is inversely proportional to the number of obstacles. A larger map size will increase the complexity of the raster map and seriously reduce the efficiency of path planning. Too many obstacles can also affect the accuracy of the planned path. Therefore, selecting the appropriate raster scale to reduce the map scale and retain the map boundary characteristics as much as possible is the key to building raster maps and improving path-planning efficiency.

The obstacle grid is determined by setting the frequency of obstacles and generating random numbers in all grids to show the effect of random obstacles in the grid map. Randomly generate a matrix of 01, where 0 represents the areas that the mobile robot can pass through, and 1 represents obstacles. After specifying the starting point and end point, the obstacle is generated with probability. Simply put, the environment is divided into a series of grids through the grid map, in which each grid is given a possible value indicating the probability that the grid is occupied. In this study, as shown in Table 6, the probability of obstacles was set at 20%, and obstacles were randomly generated on three kinds of raster maps with side lengths of 20 × 20, 30 × 30, and 40 × 40, respectively, to increase the complexity of the environmental model and further study the feasibility of the improved sparrow search algorithm.

### 5.3. Path-Planning Problem Research

**Figure 11:**
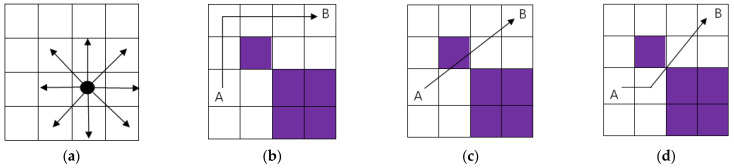
Robot walking path diagram: (**a**) travel path; (**b**) correct path; (**c**) error Path; (**d**) correct path.

A mobile robot can move on a grid whose coordinates are represented by a central point. The white grid area is the scope of the robot’s autonomous action, allowing passage. The purple grid is the obstacle area, indicating an obstacle on the road. When the robot moves to the grid in the figure, the last direction of action is removed, and it can move towards any target with contact [28], as shown in Figure 11a below. Suppose that the robot encounters obstacles from A to B, bypassing or crossing them during its movement. This can be regarded as a correct and effective path, as shown in Figure 11b,d. If there is contact with an obstacle during movement, it is regarded as an incorrect and invalid path, as shown in Figure 11c.

The robot path-planning problem is defined by a series of optimization criteria and constraints. When the traditional sparrow search algorithm is applied to the path-planning problem, its objective function is mostly based on the length of the planned path. However, under actual working conditions, the path length alone cannot be used to comprehensively evaluate the path, and the factors that determine the quality of a path are not limited to the single index of path length. Therefore, this paper selects the three indicators of path length, robot steering times, and driving bumps as the basis for the construction of the objective function [29]. The objective function is described as the weighted sum of the three values, as shown in Equation (10):

(10) $$F=\alpha \cdot F_{d}+\beta \cdot F_{s}+\gamma \cdot F_{h}$$

where $F_{d}$ is the distance factor, $F_{s}$ is the turning factor, $F_{h}$ is the elevation factor, and α, β, and γ are the weight coefficients.

(1) Distance factor

Making the length of the planned path as short as possible has long been the basic goal of path-planning research [30]. In the heuristic function of heuristic algorithms or the objective function of optimization algorithms, two methods are usually used to calculate the distance between two points: one is the Euclidean distance, and the other is the Manhattan distance. In this paper, the distance factor is defined as the shortest Euclidean distance connecting the start point to the collision-free path points of the goal point, as represented by Equation (11):

(11) $$F_{d}=min\left[\sum_{i=1}^{n-1}\sqrt{{\left(x_{i+1}-x_{i}\right)}^{2}+{\left(y_{i+1}-y_{i}\right)}^{2}}\right]$$

where $x_{i}$ is the X-coordinate of the raster corresponding to the ith path point, while $y_{i}$ is the Y-coordinate of the raster corresponding to the ith path point.

(2) Turning factors
  (12) $$F_{s}=min[\sum_{i=2}^{n-1}f_{s}]$$
  (13) $$f_{s}=\left\{\begin{array}{ll} 1, & arctan\left(\frac{y_{i}-y_{i-1}}{x_{i}-x_{i-1}}\right)\neq arctan\left(\frac{y_{i+1}-y_{i}}{x_{i+1}-x_{i}}\right)\\ 0, & else \end{array}\right.$$
  where n is the number of steering times. This method determines whether the robot turns at the point by comparing the values of the arc-tangent function corresponding to the two path points before and after: if the values of the arc-tangent function before and after are not equal, it indicates that the robot completes the turn at that point, and the number of turns at that point is recorded as 1; otherwise, the number of turns at that point is recorded as 0. The turning factor is defined as the path that has the minimum number of turning times and, thus, guides the individual sparrow to minimize the turns as much as possible, which is particularly important in complex environments where there are more obstacles.

(3) Elevation factor

In order to ensure that the robot minimizes the number of uphill and downhill movements as much as possible during the movement process [31], or that the path is more gentle, this paper combines the grid map with elevation information, introduces the elevation factor, and uses Formula (14) as follows:

(14) $$F_{h}=min[\sum_{i=1}^{n-1}|h_{i}|]$$

where h_i_ is the height of the grid corresponding to the ith path point. From the above equation, this paper determines the bumpiness of the current planned path by solving the sum of the absolute values of the heights of the grids passed by the robot. This optimization objective guides the robot to move to the position with a lower absolute value of height, which reduces the energy consumption and operation risk.

### 5.4. ISSA-Based Global Path-Planning Method for Mobile Robots

**Figure 12:**
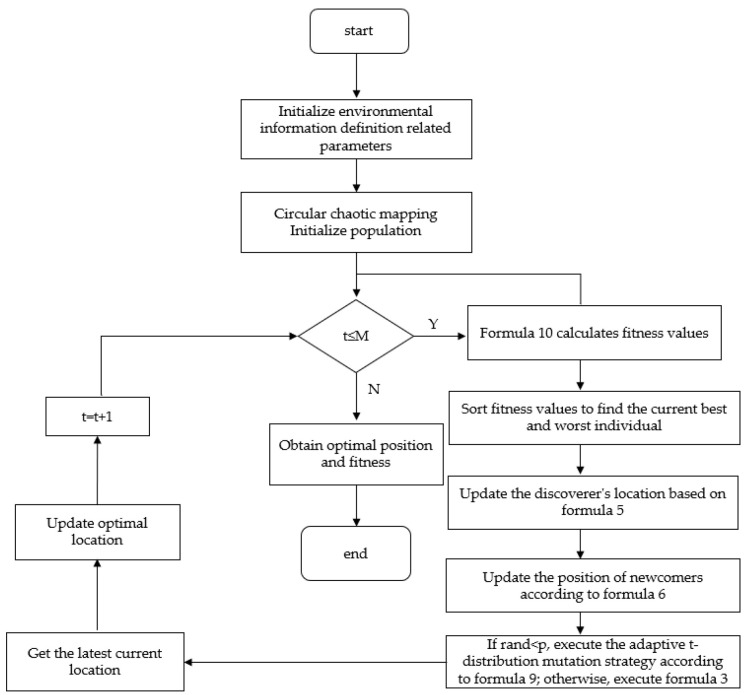
Global path-planning method based on ISSA.

From the above improved method and model, the process of applying the ISSA in path planning is shown in Figure 12.

### 5.5. Experimental Results

**Figure 13:**
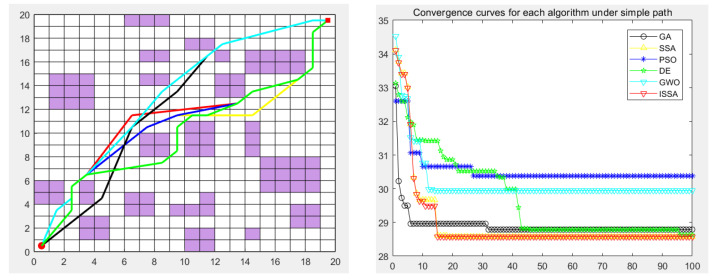
Path planning of six algorithms in environment model 1.

**Figure 14:**
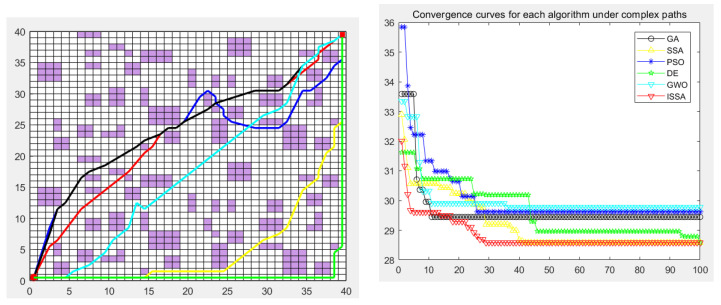
Path planning of six algorithms in environment model 2.

**Figure 15:**
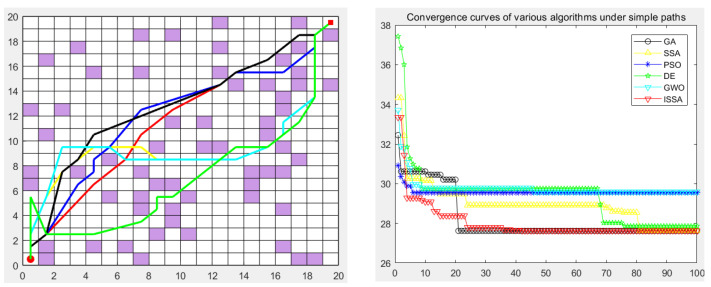
Path planning of six algorithms in environment model 3.

**Figure 16:**
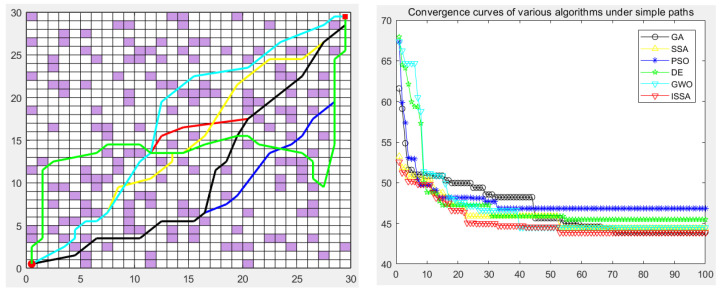
Path planning of six algorithms in environment model 4.

**Figure 17:**
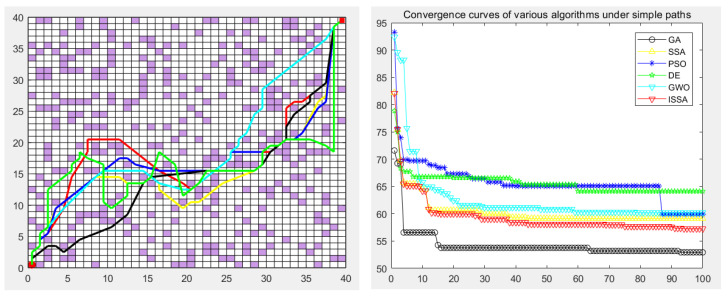
Path planning of six algorithms in environment model 5.

The genetic algorithm (GA), particle swarm optimization (PSO), differential evolution algorithm (DE), gray wolf optimization algorithm (GWO), sparrow search algorithm (SSA), and the proposed improved sparrow search algorithm (ISSA) were used to carry out the global path planning of the mobile robot in the environment model to verify the effectiveness of the ISSA. The GA, PSO, DE, GWO, SSA, and ISSA were used to solve the objective function of the modified path-planning model, finding that more paths are needed. In the simulation experiment, the population size of the GA, PSO, DE, GWO, SSA and ISSA was set to 50, the maximum number of iterations was set to 100, and 30 simulation experiments were carried out. Each individual is a path, as shown in Figure 13, Figure 14, Figure 15, Figure 16 and Figure 17, which detail the path planning of the six algorithms in the five model maps and the iterative convergence curves of the six algorithms.

In the simulation experiment of path planning for the environmental model, the GA, PSO, DE, GWO, SSA, and ISSA were used to plan the global path of the mobile robot [32]. Among them, for the path-planning simulation results, the black line represents the genetic algorithm (GA), the yellow line represents the sparrow search algorithm (SSA), the blue line represents the particle swarm optimization (PSO), the green line represents the differential evolution algorithm (DE), and the green line represents the gray wolf algorithm (GWO). Red represents the improved sparrow search algorithm (ISSA).

The experimental results show that the ISSA can accurately find the shortest path in two different maps. By comparing the results of Figure 13 and Figure 14, we can see that the fusion strategy of the improved sparrow search algorithm (ISSA) achieved good results on the shortest path in both selected maps. In environment model 1, the SSA can quickly plan a better path from the starting point to the target point. However, for environment model 2, although the six algorithms all plan the target path, the path planned by the DE algorithm and SSA has many turning points, the path is not smooth enough, and the length is not short enough, which increases the cost of mobile robot path planning and consumes time. In some other performance indicators, the six algorithms show different advantages, and it is in these specific evaluation indicators that the advantages of the ISSA are more obvious. It not only has the fastest convergence speed, but also has the highest planning success rate, which fully reflects the high efficiency and excellent performance of the ISSA.

The performance of each algorithm can be further analyzed from the experimental results. For map model 1 with simple obstacle distribution (as shown in Figure 9), although algorithms such as the GA, PSO, DE, GWO, SSA, and ISSA [33] have specific differences in other evaluation indicators, these algorithms can find the optimal path almost every time they run. The ISSA can obtain a path with a shorter length and better smoothness than the other five algorithms. The results obtained by the ISSA in the path-planning experiment show little difference, which verifies the stability of this algorithm in practical application. At the same time, as seen in Figure 13 and Figure 14, the path planned by the ISSA successfully avoids environmental obstacles and successfully reaches the target area. The results of ISSA path planning under the two models were compared with those of other algorithms. The results show that the ISSA is effective in global path planning for mobile robots.

In order to verify the validity of the proposed ISSA, in the path planning of maps 3, 4, and 5 of the random-obstacle environment, the experimental results show that the ISSA can accurately find the shortest path in three maps of different complexity. By comparing the results of Figure 15 and Figure 16, we can see that the ISSA improved by the fusion strategy achieves good results on the shortest path in both selected maps. The GWO, DE, and SSA also successfully planned the path to the target point, but the planned path was more complex, and the cost of the optimal path length, node, or search time was higher. For Figure 15, although the effect of the path and convergence curve planned by the ISSA is worse than that of the GA, compared with the other four algorithms, it can be seen that the route planned by the ISSA is better than that of the original algorithm. It can plan a path without collision obstacles from the starting point to the end point, and the path length is shorter. Its solving performance can be improved by improving the multi-strategy of the SSA, and a path with a shorter length can be planned.

The experimental results show that we can analyze the performance of each algorithm in depth. In the simple map scene with a clear obstacle layout, as shown in Figure 15, various algorithms, such as the GA, PSO, DE, GWO, SSA, and ISSA, can find the best path almost every time they run, despite slight differences in other evaluation indicators. However, in the large map presented in Figure 16, which contains many complex obstacles and traps, the SSA and the DE algorithm need help finding the shortest path, while the ISSA can still find the optimal path. In this case, we need to be aware that it may take more optimization time or more iterations to find the global optimal path in the face of a larger map size. It is worth noting that although the ISSA’s convergence rate is the fastest in Figure 15, it is second only to the GA’s when facing large-scale raster maps (Figure 17). However, considering that the optimal path can be found even in these two maps, we can still assume that the convergence rate of the ISSA is relatively fast.

In summary, in the actual scenario of mobile robot path planning, the robot may need to adjust the path in real time to cope with environmental changes or new task requirements. Through the ISSA, we can quickly replan the path and perform well in real time. This means that the robot can respond in time, adapt to new situations, and maintain an efficient working state.

In this chapter, an improved sparrow search algorithm (ISSA) was proposed, which was applied to solve the path-planning problem, and the advantages of this algorithm in solving the path-planning problem were verified through experiments. Firstly, we designed two groups of experiments with different sizes of search space in 20 × 20 and 40 × 40 raster maps, and then we experimented with three groups of random-obstacle raster maps in 20*20, 30 × 30, and 40 × 40 raster maps, and compared them with other swarm intelligence algorithms, showing that the optimal path length of the improved sparrow search algorithm compares favorably with that of other swarm intelligence optimization algorithms. The optimal path length, number of inflection points, and search time were all reduced. This shows that the ISSA has relatively fast convergence speed and high stability, which verifies that it is effective when applied to the global path-planning problem of mobile robots. However, there are still some shortcomings in this experiment; global path planning is highly dependent on an accurate environment map, along with insufficient flexibility, poor adaptability to environmental changes, and other problems. Overall, the practical application of global path planning for mobile robots demonstrates the importance of intelligent decision-making, safety and reliability, optimized efficiency, application domain expansion, and flexibility and adaptability. These insights suggest that we should focus on considering the intelligence, safety, efficiency, and adaptability of algorithms when designing and applying intelligent systems in order to promote the wide application and development of automation technology in various fields.

## 6. Conclusions

Path planning is crucial for mobile robots and determines how efficiently and safely the robot can reach the target location. Global path planning pursues the optimal path, while local path planning ensures real-time obstacle avoidance. Given the advantages of intelligent bionic algorithms in global planning, this paper makes innovative improvements based on the sparrow search algorithm and presents a practical mobile robot path-planning method to enhance the robot’s task execution efficiency and environmental adaptability.

In this paper, an improved sparrow search algorithm is proposed for the problems of poor optimization effect and low search stability in mobile roaming robot path planning. By testing the 10 standard test functions of CEC2021 and comparing it with seven other swarm intelligence algorithms, the comparative analysis of the experimental simulation results showed that the ISSA is able to break through the local optimal solution, obtains higher accuracy, and has a stronger global search capability compared with other schemes. Applying it to solve the path-planning problem, two groups of experiments with different search space sizes were first designed, and the performance and stability of the improved algorithm were verified by changing the complexity of the map. Then, through three sets of random-obstacle raster maps, the experimental results show that the improved sparrow search algorithm reduces the optimal path length, the number of inflection points, and the search time, compared with other swarm intelligence optimization algorithms in the raster maps. The improved algorithm has better performance in optimal path length, number of nodes, and search time, reflecting that the ISSA has a faster convergence speed and higher stability, which verifies that its application to the global path-planning problem of mobile robots is effective.

In this paper, an improved sparrow search algorithm is proposed for global path planning of robots, modeled by the 2D raster method. Experiments verified the excellent performance of the algorithm in solving this problem, as well as its practicality. However, the following shortcomings still exist: (1) This study mainly focuses on simulating the horizontal movement scenario, and we did not consider the three-dimensional movement condition of UAVs. Therefore, future research should conduct experiments in three-dimensional space to expand the application scope of the algorithm. (2) This paper was mainly based on simulation experiments, and future research should be carried out in real environments to broaden the practical application areas of the algorithm.

## Figures and Tables

**Table 1:** Parameter settings.

Arithmetic	Parameterization
SSA	PD = 20%, R_2_ = 0.8, SD = 10%
ISSA	PD = 20%, R_2_ = 0.8, SD = 10%, ω_max_ = 1, ω_min_ = 0.4
DBO	γ = 0.1, k = 0.1, u = 0.3, s = 0.5
GWO	a decreases from 2 to 0
NGO	R = 0. 02 × (1 − t/Maxiter), w = 0.9
PSO	*w*_max_ = 0.9, *w*_min_ = 0.4, *c*_1_ = *c*_2_ = 2
CASSA	PD = 20%, R_2_ = 0.8, SD = 10%, ω_max_ = 1, ω_min_ = 0.4
YDSE	I = 0.01, L = 1, d = 5 × 10^−3^

**Table 2:** CEC2021 test function.

	No.	Function	FI *
Unimodal Function	1	Shifted and Rotated Bent Cigar Function (CEC 2017 [4] F1)	100
Basic Functions	2	Shifted and Rotated Schwefel’s Function (CEC 2014 [3] F11)	1100
	3	Shifted and Rotated Lunacek bi-Rastrigin Function (CEC 2017 [4] F7)	700
	4	Expanded Rosenbrock’s plus Griewangk’s Function (CEC2017 [4] f_19_)	1900
Hybrid Functions	5	Hybrid Function 1 (N = 3) (CEC 2014 [3] F17)	1700
	6	Hybrid Function 2 (N = 4) (CEC 2017 [4] F16)	1600
	7	Hybrid Function 3 (N = 5) (CEC 2014 [3] F21)	2100
Composition Functions	8	Composition Function 1 (N = 3) (CEC 2017 [4] F22)	2200
	9	Composition Function 2 (N = 4) (CEC 2017 [4] F24)	2400
	10	Composition Function 3 (N = 5) (CEC 2017 [4] F25)	2500
Search range: [−100, 100]D			

FI * denotes the optimal value of the test function.

**Table 3:** Comparison among optimal results of benchmarking functions.

		ISSA	SSA	DBO	YDSE	GWO	PSO	NGO	HHO
F1	min	0	0	0	0.015284	2.4 × 10^−118^	3.04 × 10^−18^	1.9 × 10^−200^	9.1 × 10^−212^
	std	0	1.86 × 10^−68^	0	0.200305	3.5 × 10^−111^	2537.081	0	0
	avg	0	3.39 × 10^−69^	2.3 × 10^−223^	0.250047	1.3 × 10^−111^	666.6667	5.1 × 10^−195^	8.8 × 10^−177^
	median	0	7.44 × 10^−79^	1.9 × 10^−262^	0.193235	4.8 × 10^−114^	1.2 × 10^−16^	9.9 × 10^−197^	2.5 × 10^−193^
	worse	0	1.02 × 10^−67^	6.8 × 10^−232^	0.789163	1.5 × 10^−110^	10000	6 × 10^−194^	2.6 × 10^−175^
F2	min	0	0	0	19.02603	0	0.249818	0	0
	std	0	0	0.022805	186.9538	4.064476	116.2454	0.087062	0
	avg	0	0	0.004164	388.8823	2.143601	99.71918	0.027247	0
	median	0	0	0	354.8508	9.09 × 10^−13^	66.23645	0	0
	worse	0	0	0.124909	790.2157	15.18306	458.4334	0.380224	0
F3	min	0	0	0	18.2813	0	0.994959	0	0
	std	0	0	16.99432	4.573419	17.24524	5.390551	4.14 × 10^−27^	0
	avg	0	0	9.343137	25.28394	31.76978	10.4188	7.56 × 10^−28^	0
	median	0	0	0	25.47547	36.66364	12.14196	0	0
	worse	0	0	54.41579	35.16091	64.03087	21.03211	2.27 × 10^−26^	0
F4	min	0	0	0	0.696801	0	0.42937	0	0
	std	0	0	0.838748	0.315215	0.564758	0.19892	0.035287	0
	avg	0	0	0.562159	1.250387	0.336316	0.713897	0.009235	0
	median	0	0	0	1.200206	0.020998	0.668404	0	0
	worse	0	0	2.666209	1.957046	2.058048	1.139975	0.150512	0
F5	min	0	0	2.3 × 10^−261^	14.86381	3.34 × 10^−96^	0.416286	4.2 × 10^−27^	6.7 × 10^−207^
	std	0	3.24 × 10^−16^	1.86 × 10^−19^	14.30704	0.529293	152.4478	8.94 × 10^−25^	0
	avg	0	6.82 × 10^−17^	3.79 × 10^−20^	37.92861	0.166304	181.6502	2.65 × 10^−25^	2.4 × 10^−173^
	median	0	9.61 × 10^−19^	4.83 × 10^−28^	35.9939	1.26 × 10^−31^	163.1174	8.22 × 10^−26^	5.9 × 10^−188^
	worse	0	1.78 × 10^−15^	1.02 × 10^−18^	69.52328	2.227112	526.4074	4.96 × 10^−24^	5.5 × 10^−172^
F6	min	0	0	0	1.852781	2.23 × 10^−05^	0.244513	0.000346	0
	std	0	2.49 × 10^−06^	0.390172	7.751756	1.403388	21.78618	0.064971	5.06 × 10^−05^
	avg	0	1.12 × 10^−06^	0.135026	10.24319	0.513367	7.46933	0.031283	1.72 × 10^−05^
	median	0	2.11 × 10^−08^	1.7 × 10^−06^	8.578803	0.043709	0.997865	0.023131	1.33 × 10^−09^
	worse	0	1.05 × 10^−05^	1.319029	40.33987	6.801991	118.7575	0.368195	0.000256
F7	min	0	0	2.5 × 10^−118^	0.616441	2.26 × 10^−05^	0.02437	7 × 10^−05^	3.7 × 10^−214^
	std	0	0.001786	0.304532	4.882744	0.258574	58.11001	0.000579	8.65 × 10^−06^
	avg	2.5 × 10^−279^	0.000354	0.073356	5.604158	0.083544	56.92354	0.000628	1.74 × 10^−06^
	median	1.6 × 10^−305^	2.23 × 10^−06^	8.67 × 10^−07^	4.549893	0.011282	17.48157	0.000455	6.02 × 10^−15^
	worse	7.4 × 10^−264^	0.009806	1.623121	26.92456	1.215745	136.089	0.002573	4.74 × 10^−05^
F8	min	0	0	0	16.45564	0	5.18 × 10^−15^	0	0
	std	0	0	0	77.82427	0	32.68747	0	0
	avg	0	0	0	78.08676	0	23.15519	0	0
	median	0	0	0	55.60085	0	20.72099	0	0
	worse	0	0	0	332.8596	0	184.3961	0	0
F9	min	0	8.2 × 10^−148^	3.2 × 10^−307^	0.011849	8.88 × 10^−15^	8.07 × 10^−12^	1.28 × 10^−86^	1.3 × 10^−211^
	std	0	2.34 × 10^−66^	0	0.038802	0	1.33 × 10^−10^	1.62 × 10^−15^	0
	avg	0	4.27 × 10^−67^	1.2 × 10^−166^	0.057248	8.88 × 10^−15^	1.04 × 10^−10^	8.59 × 10^−15^	2.6 × 10^−187^
	median	0	1.07 × 10^−81^	6.3 × 10^−270^	0.046869	8.88 × 10^−15^	6.29 × 10^−11^	8.88 × 10^−15^	2.5 × 10^−200^
	worse	0	1.28 × 10^−65^	3.6 × 10^−165^	0.187565	8.88 × 10^−15^	6.8 × 10^−10^	8.88 × 10^−15^	7.8 × 10^−186^
F10	min	0	1.5 × 10^−222^	6.18 × 10^−11^	48.68945	48.91734	0.01106	0.000838	9.8 × 10^−210^
	std	0	8.51 × 10^−05^	26.71263	0.277585	10.87702	10.38203	16.89705	0.00023
	avg	0	5.82 × 10^−05^	21.28953	49.33463	55.8605	48.04923	6.51828	8.46 × 10^−05^
	median	0	6.51 × 10^−10^	0.001958	49.29573	50.1522	48.37445	0.001947	3.16 × 10^-07^
	worse	0	0.000309	67.07167	49.82168	78.9468	75.79175	49.04756	0.00119

**Table 4:** Comparison between the best results of three algorithmic test functions.

		Min	Std	Avg	Median	Worse
F1	ISSA	0	0	0	0	0
	SSA	5.20 × 10^−135^	6.91 × 10^−09^	1.36 × 10^−69^	2.72 × 10^−76^	3.78 × 10^−68^
	CASSA	2.01 × 10^−124^	9.68 × 10^−40^	1.76 × 10^−40^	2.06 × 10^−58^	5.30 × 10^−39^
F2	ISSA	0	0	0	0	0
	SSA	0	2.77 × 10^−13^	9.09 × 10^−14^	0	9.09 × 10^−13^
	CASSA	0	0	0	0	0
F3	ISSA	0	0	0	0	0
	SSA	0	0	0	0	0
	CASSA	0	0	0	0	0
F4	ISSA	0	0	0	0	0
	SSA	0	0	0	0	0
	CASSA	0	0	0	0	0
F5	ISSA	0	0	0	0	0
	SSA	1.22 × 10^−251^	2.79 × 10^−17^	1.10 × 10^−17^	5.26 × 10^−21^	1.11 × 10^−16^
	CASSA	3.50 × 10^−66^	4.93 × 10^−17^	9.55 × 10^−18^	1.33 × 10^−23^	2.70 × 10^−16^
F6	ISSA	0	0	0	0	0
	SSA	0	2.65 × 10^−06^	1.96 × 10^−06^	7.22 × 10^−07^	1.02 × 10^−05^
	CASSA	0	9.15 × 10^−14^	2.27 × 10^−14^	1.23 × 10^−29^	4.54 × 10^−13^
F7	ISSA	0	0	4.48 × 10^−271^	0	1.25 × 10^−269^
	SSA	0	0.002619909	0.00070175	7.09231 × 10^−07^	0.010439418
	CASSA	9.53 × 10^−150^	4.38 × 10^−20^	8.56 × 10^−21^	3.64 × 10^−38^	2.40 × 10^−19^
F8	ISSA	0	0	0	0	0
	SSA	0	0	0	0	0
	CASSA	0	0	0	0	0
F9	ISSA	0	0	1.69 × 10^−299^	0	5.07 × 10^−298^
	SSA	0	4.78 × 10^−65^	8.99 × 10^−66^	8.61 × 10^−87^	2.62 × 10^−64^
	CASSA	7.65 × 10^−120^	3.94 × 10^−43^	7.19 × 10^−44^	1.64 × 10^−66^	2.15 × 10^−42^
F10	ISSA	0	0	0	0	0
	SSA	3.10 × 10^−96^	0.000151407	9.89 × 10^−05^	5.72 × 10^−11^	0.000451946
	CASSA	2.74 × 10^−138^	1.29 × 10^−15^	2.36 × 10^−16^	2.56 × 10^−63^	7.10 × 10^−15^

**Table 5:** Comparison of ablation experiment results.

		Min	Std	Avg	Median	Worse
F3	NGOSSA	0	0	0	0	0
	CirSSA	0	0	0	0	0
	LeySSA	0	0	0	0	0
	SSA	0	3.60 × 10^−32^	6.57 × 10^−33^	0	1.97 × 10^−31^
	ALSSA	0	0	0	0	0
F4	NGOSSA	0	0	0	0	0
	CirSSA	0	0	0	0	0
	LeySSA	0	0	0	0	0
	SSA	0	0	0	0	0
	ALSSA	0	0	0	0	0
F6	NGOSSA	0	5.58 × 10^−06^	2.26 × 10^−06^	9.84 × 10^−08^	2.34 × 10^−05^
	CirSSA	0	4.42 × 10^−06^	2.35 × 10^−06^	1.49 × 10^−08^	1.81 × 10^−05^
	LeySSA	0	2.08 × 10^−05^	5.23 × 10^−06^	1.01 × 10^−07^	0.000114711
	SSA	3.37 × 10^−05^	0.000926195	0.00101274	0.000749823	0.003835879
	ALSSA	0	2.08 × 10^−05^	6.35 × 10^−06^	2.39 × 10^−07^	0.000113873
F8	NGOSSA	0	0	0	0	0
	CirSSA	0	0	0	0	0
	LeySSA	0	0	0	0	0
	SSA	0	2.81 × 10^−16^	7.40 × 10^−17^	0	1.11 × 10^−15^
	ALSSA	0	0	0	0	0
F9	NGOSSA	5.33 × 10^−168^	6.37 × 10^−63^	1.16 × 10^−63^	1.44 × 10^−92^	3.49 × 10^−62^
	CirSSA	0	3.65 × 10^−70^	6.66 × 10^−71^	8.79 × 10^−94^	2.00 × 10^−69^
	LeySSA	1.70 × 10^−191^	5.87 × 10^−70^	1.49 × 10^−70^	2.38 × 10^−88^	2.84 × 10^−69^
	SSA	6.02 × 10^−40^	6.84 × 10^−15^	3.55 × 10^−15^	3.82 × 10^−35^	2.66 × 10^−14^
	ALSSA	1.54 × 10^−126^	2.50 × 10^−63^	4.57 × 10^−64^	8.27 × 10^−85^	1.37 × 10^−62^

**Table 6:** Random-obstacle environment map.

Raster Map Name	Dimension	Number of Grids	Percentage of Obstacles	Number of Obstacle Grids
Raster map 4	20 × 20	400	20%	80
Raster map 5	30 × 30	900	20%	180
Raster map 6	40 × 40	1600	20%	320

## Data Availability

The original contributions presented in the study are included in the article, further inquiries can be directed to the corresponding author.
